# Predictors of Heart Failure in Pediatric Patients with End-Stage Kidney Disease Secondary to Nephrotic Syndrome

**DOI:** 10.3390/medicina62061131

**Published:** 2026-06-10

**Authors:** Meng Wei, Huiping Huang, Yajun Shen, Li Wei, Yifei Li, Hui Guo

**Affiliations:** 1 Ministry of Education Key Laboratory of Women and Children’s Diseases and Birth Defects, Department of Pediatrics, West China Second University Hospital, Sichuan University, Chengdu 610000, China; weimeng000124@stu.scu.edu.cn (M.W.); shenyajun0828@scu.edu.cn (Y.S.); weili1010@scu.edu.cn (L.W.); 2 Department of Pediatrics, The First People’s Hospital of Shuangliu District, West China Airport Hospital of Sichuan University, Chengdu 610207, China; 19108056627@163.com

**Keywords:** end stage kidney disease, cardiorenal syndrome, risk factors, prognosis

## Abstract

*Background and Objectives*: To investigate prognostic risk factors and determine the incidence, clinical characteristics, and predictors of heart failure (HF) development in pediatric patients with end-stage kidney disease (ESKD) secondary to steroid-resistant nephrotic syndrome (SRNS). *Materials and Methods*: We conducted a retrospective cohort study of pediatric patients diagnosed with ESKD secondary to nephrotic syndrome (NS) between 2014 and 2020. Patients were stratified based on clinical outcomes and the occurrence of HF during follow-up. Comparative analyses of clinical characteristics, laboratory parameters, and cardiac assessments were performed across groups. Multivariate logistic regression was used to identify independent risk factors for HF development within the first year and for adverse prognosis at five years. *Results*: The cohort comprised 172 children with ESKD secondary to NS. Multivariate logistic regression identified HF as an independent risk factor for adverse long-term outcomes in pediatric patients with ESKD. During follow-up, HF developed in 27 patients (15.7%) within the first year after ESKD diagnosis, and in 45 patients (26.2%) by the end of five years. Early HF onset (within the first year) was associated with a significantly reduced five-year survival rate. Independent risk factors for HF development included elevated cardiac troponin I levels (OR = 6.786, 95% CI: 2.326–19.799), a history of cardiac arrhythmias (OR = 2.951, 95% CI: 1.260–6.912), and the presence of left heart enlargement (OR = 23.669, 95% CI: 2.876–194.827), and valvular regurgitation at the initial post-ESKD diagnosis evaluation. *Conclusions*: HF is associated with markedly reduced survival. Crucially, our findings demonstrate that pre-existing cardiovascular structural abnormalities—specifically left heart enlargement—and elevated cTnI are robust, early predictors of HF. These findings necessitate a paradigm shift in pediatric ESKD management, we advocate for the implementation of systematic baseline echocardiographic and biomarker screening at the immediate onset of ESKD. Identifying these subclinical, yet modifiable, structural changes provide a critical therapeutic window for targeted anti-remodeling interventions to significantly improve long-term prognosis.

## 1. Introduction

Chronic kidney disease (CKD) is defined as abnormalities in kidney structure or function persisting for at least three months with implications for health [1]. CKD encompasses a heterogeneous group of disorders that progressively and irreversibly compromise renal function and structure through distinct pathophysiological pathways over months to years, ultimately culminating in end-stage kidney disease (ESKD) [2]. CKD is a growing global health concern, and the rapid increase in its overall incidence and prevalence among children and adolescents worldwide requires greater attention [3,4].

Nephrotic syndrome (NS), especially steroid-resistant NS (SRNS), has been identified as one of the top causes of ESKD in pediatric patients [5,6]. The association between pediatric NS and progression to ESKD represents a complex pathophysiological continuum with significant clinical implications for long-term patient outcomes. The underlying histopathological subtype serves as a critical determinant of renal prognosis [7]. The pathogenetic mechanisms underlying NS progression to ESKD involve multiple interconnected pathways, including persistent proteinuria-induced tubular toxicity, activation of inflammatory cascades, progressive glomerulosclerosis, and tubulointerstitial fibrosis [8]. A core pathological feature of SRNS is profound podocyte injury and the disruption of the glomerular filtration barrier. Whether driven by genetic defects encoding crucial slit diaphragm structural proteins or through acquired dysregulation of the complex podocyte cytoskeleton, the ultimate consequence is irreversible foot process effacement. This profound structural collapse results in massive, persistent proteinuria, serving as the primary catalyst accelerating the rapid progression from CKD to ESKD [9]. Contemporary therapeutic strategies, including the implementation of renin-angiotensin system blockade, immunosuppressive regimens, and aggressive blood pressure management, have demonstrated efficacy in attenuating disease progression and preserving residual renal function [10]. However, while most children with steroid-sensitive NS achieve a favorable prognosis through standardized treatment, those with SRNS face a significantly higher risk of progressing to ESKD [6]. A substantial proportion of these patients require the initiation of renal replacement therapy (RRT) as early as childhood or adolescence [11]. Early identification of high-risk patients through comprehensive histopathological evaluation, genetic testing for hereditary nephropathies, cardiac function evaluation and serial monitoring of renal function parameters remains paramount for optimizing therapeutic interventions and improving long-term outcomes in this SRNS associated ESKD population [12].

Cardiovascular disease is of particular clinical significance as it represents the leading cause of morbidity and mortality in CKD patients, manifesting as coronary artery disease, heart failure (HF), cardiac arrhythmias, and sudden cardiac death, with risk escalating dramatically as patients progress toward ESKD [13,14]. The pathophysiological continuum characterized by cardiac injury or dysfunction secondary to chronic kidney disease is clinically recognized as type 4 cardiorenal syndrome (CRS) [15]. Generally, the incidence of de novo HF is 25–40% in such ESKD patient population. The development of HF in patients with ESKD represents a critical prognostic determinant that substantially increases morbidity and mortality risk through complex bidirectional pathophysiological interactions [13]. From a prognostic perspective, HF in ESKD patients is associated with increased hospitalization rates, reduced quality of life, and significantly decreased survival, with median survival ranging from 1 to 3 years following HF diagnosis. In adults, the severity of proteinuria and hypertension caused by Nephrotic Syndrome (NS) are recognized high-risk factors for cardiovascular disease and poor overall prognosis [16]. However, pediatric studies have yielded conflicting results, with some supporting [17] and others failing to demonstrate this association [18]. Furthermore, regarding etiology, adult CKD is predominantly caused by diabetes mellitus and other acquired conditions, whereas pediatric CKD primarily results from congenital anomalies [19]. Consequently, extrapolating conclusions from adult data to pediatric populations requires careful consideration. Specifically, the predictors and progression of cardiorenal syndrome in children with ESKD, particularly those secondary to nephrotic syndrome, remain inadequately defined. Therefore, in this study, we conducted a retrospective analysis on the clinical data, cardiac assessments, and attempted to conclude a correlation with all-cause mortality.

## 2. Methods

### 2.1. Patient Population

This single-center, retrospective cohort study was conducted at West China Second University Hospital, Sichuan University, with patient enrollment spanning from January 2014 to December 2020. The study design adhered strictly to the Strengthening the Reporting of Observational Studies in Epidemiology (STROBE) statement guidelines to ensure methodological rigor and transparent reporting. The study protocol received approval from the Ethics Committee of West China Second Hospital of Sichuan University (approval number: 2021-073). All parents or legal guardians of patients have signed an informed consent form, agreeing to use their children’s clinical and imaging data for the publication of this article. Two trained physicians retrospectively extracted data from electronic medical records and follow-up databases. To ensure accuracy, all data underwent independent double-verification and cross-validation.

The minimum follow-up duration was established at five years or until death, whichever occurred first, to provide adequate observation time for assessment of long-term outcomes and prognostic factors. This extended follow-up period enables comprehensive evaluation of disease progression and identification of clinically relevant endpoints in the study population.

### 2.2. Inclusion and Exclusion Criteria

Inclusion criteria were as follows: (1) all patients met the diagnostic criteria for ESKD. In this study, the estimated glomerular filtration rate (eGFR) was calculated using the Bedside Schwartz formula. Inclusion required a sustained eGFR below 15 mL/min/1.73 m^2^, and all patients were receiving scheduled, regular maintenance RRT; (2) the ESKD in all patients developed from preexisting NS, and NS had been in complete remission when ESKD diagnosis; (3) the previous treatment of NS demonstrated a typical procedure of SRNS; (4) at the time of initial ESKD diagnosis, all patients were free of heart failure and identifiable cardiovascular injury, as evidenced by preserved left ventricular systolic function, defined as an ejection fraction (EF ≥ 55%) and fractional shortening (FS ≥ 25%); (5) cardiac assessment was completed within the first year after initially meeting the aforementioned diagnostic criteria for ESKD; (6) the follow-up duration remained at least for 5 years or until death.

The exclusion criteria included the following: (1) patients demonstrated any congenital cardiovascular or renal malformation; (2) patients had a pre-existing diagnosis of a systemic autoimmune disease (e.g., systemic lupus erythematosus, ANCA-associated vasculitis) before the onset of NS; (3) any cancer or tumor history; (4) syndromic genetic nephropathies including inherited NS and any identified genetic variants with inherited cardiovascular diseases; (5) incomplete cardiac assessments; (6) received renal transplantation within 5 years as we did not set renal transplantation as an end point of follow-up; (7) immunodeficiency due to non-renal factors (e.g., HIV infection or genetic disorders); (8) lost to follow-up due to various reasons during the follow-up period.

### 2.3. Therapeutic and Follow-Up Procedures

All patients were diagnosed with NS at the onset of the disease and received standard steroid therapy in accordance with the KDIGO guidelines. Once ESKD was diagnosed, RRT was initiated per guidelines, and the option for renal transplantation was discussed with the family. Additional treatments were provided for associated complications, including infection, anemia, hypertension, HF, mineral and bone disorder with ESKD. The therapeutic strategy for HF included angiotensin-converting enzyme inhibitors (ACEI), angiotensin receptor blockers (ARB), β-blocker, sodium-glucose cotransporter-2 inhibitor (SGLT2i), angiotensin receptor-neprilysin inhibitor (ARNi) and digoxin, which were administered at full doses under routine RRT. These therapeutic strategies represent standard good clinical practice and were not part of the specific study design.

All the enrolled patients received scheduled RRT or peritoneal dialysis. RRT received from external hospital was also acceptable and kept for follow-up. The primary outcome was designed as all-cause mortality and we evaluated whether HF contributed to higher all-cause mortality of pediatric ESKD. Accordingly, patients were subdivided into survivors and non-survivors at five years. And the second outcome was cardiac dysfunction events, while subgroup division was formed to assess the potential risk factors for HF. All the involved patients were required to receive echocardiographic evaluations within 2 weeks post ESKD diagnosed, and cardiac assessment would complete every year during follow-up.

### 2.4. Echocardiographic Evaluation and Clinical Data Collection

All echocardiographs were performed by two well-trained pediatric physicians. The physicians involved in the examination of enrolled patients were blinded to the clinical manifestation of receivers. The first echocardiography was performed within 2 weeks post-ESKD diagnosis. Annual echocardiography was performed during follow-up. We defined the morphology and function according to the reference data equalized to their height and weight. Echocardiographic evaluation encompassed structural parameters (left ventricular internal diameter, cardiac dimensions, interventricular septal and posterior wall thickness, pulmonary and ascending aortic diameters), valvular assessments (aortic, mitral, and tricuspid regurgitation), hemodynamic measurements (pulmonary artery flow, atrial shunting, pulmonary hypertension), and pathological findings (pericardial effusion, coronary artery dilation).

In this paper, HF was defined as a left ventricular ejection fraction (EF) below 55% and/or a fractional shortening (FS) below 25%. The phenotype of HF occurring within the first year after a confirmed diagnosis of ESKD is defined as aggressive HF. The definitions of left ventricular/whole heart dilation, ventricular hypertrophy, vascular dilation, and pathological valvular regurgitation were strictly based on international guidelines and authoritative studies in the field of pediatric cardiology. Cardiac enlargement and ventricular hypertrophy were primarily defined based on “Z-score standardization,” while vascular dilation was assessed using absolute internal diameters and relative ratios. The severity of aortic, mitral, and tricuspid regurgitation was evaluated semi-quantitatively and categorized as trace, mild, moderate, or severe, ensuring the scientific validity and pediatric specificity of the criteria. During data collection, regurgitation was considered pathological and clinically significant if it was moderate or severe. Left ventricular dilation was defined as a standardized Z-score of left ventricular end-diastolic dimension (LVEDD) > 2.0; whole heart enlargement was defined as Z-scores > +2 in multiple cardiac chambers, such as both the left and right ventricles; ventricular hypertrophy was defined as a Z-score > +2 for either interventricular septal thickness or left ventricular posterior wall thickness; vascular dilation was defined as an internal diameter exceeding the upper limit of normal for the corresponding age.

Clinical data were systematically collected at the time of initial hospitalization for ESKD diagnosis, encompassing baseline demographic characteristics (age, gender, systolic blood pressure, presence of edema), comprehensive medical history including hypertension, hypertensive encephalopathy, cardiac arrhythmias, renovascular hypertension, secondary hyperparathyroidism, pulmonary infections, anemia, thrombotic events, metabolic acidosis, hyperuricemia, and relevant family history, as well as treatment modalities comprising RRT and peritoneal dialysis. Laboratory parameters included renal and electrolyte assessments [glomerular filtration rate (GFR), potassium, chloride, calcium, phosphorus, and glucose levels], hematological parameters [erythropoietin (EPO), eosinophil count (EOS), neutrophil count (NEUT), mean corpuscular hemoglobin (MCH), and platelet count (PLT)], hepatic and cardiac biomarkers [globulin (GLB), aspartate aminotransferase (AST), alanine aminotransferase (ALT), cardiac troponin I (cTnI), creatine kinase-MB (CK-MB), N-terminal pro-brain natriuretic peptide (NT-proBNP), and myoglobin (Mb)], coagulation studies [international normalized ratio (INR), D-dimer (DDI), fibrin (ogen) degradation products (FDP), and antithrombin III (ATIII)], and immunological markers [CD3^+^CD8^+^ T-cell subsets, high-sensitivity C-reactive protein (hs-CRP), immunoglobulin E (IgE), and immunoglobulin G (IgG)].

### 2.5. Risk Factor Analysis

At first, the basic clinical characteristics and hematological examination results of the enrolled patients were recorded, and all the parameters are listed in Table 1. Then, univariate analysis was performed between the patients between survival and dead patients in 5 years follow-up. Then, the multivariable analysis was completed using logistic regression to identify the independent factors among the significant results according to univariable analysis. In addition, all parameters had been used to evaluate the risk factors for HF, especially for echocardiographic results. Given the large number of comparisons involved in univariate analysis, in order to control for the risk of false positives caused by multiplicity, we used the Benjamini–Hochberg program to correct for false discovery rate (FDR) on the *p*-values of all univariate analyses. Afterward, receiver operating characteristic (ROC) curves were used to identify the predictive value of the risk factors that were found by comparison.

### 2.6. Bias

To address potential sources of bias, we implemented the following measures: (1) We used predefined, objective inclusion and exclusion criteria to minimize selection bias. (2) To reduce information bias, all echocardiographic assessments were performed by pediatric cardiologists who were blinded to the patients’ clinical outcomes. (3) Data abstraction was conducted independently by two trained physicians with subsequent cross-verification. (4) We controlled for potential confounding by adjusting for key covariates (age, sex, baseline eGFR, hypertension) in multivariable logistic regression models. (5) An active follow-up protocol was employed to minimize loss to follow-up, and the minimum follow-up duration was set at 5 years.

### 2.7. Statistical Analysis

Data analysis was conducted using SPSS 22.0 (SPSS Inc. Chicago, IL, USA). Quantitative data were presented as the mean ± standard deviation and median with range, while qualitative data were expressed as n. Differences between the two groups were assessed using the independent *t* test or Mann–Whitney U test for continuous variables and the chi-squared test or Fisher’s exact test for categorical variables. Missing data were minimal <5% for any key variable (Supplementary Table S1), and were assumed to be missing completely at random; thus, only complete cases were included in the analyses. To estimate the cumulative incidence of all-cause mortality and heart failure (HF) during follow-up, Kaplan–Meier survival curves were constructed, and comparisons between groups were performed using the log-rank test. To identify independent predictors associated with the overall occurrence of 5-year all-cause mortality as a binary outcome, multivariable logistic regression was employed. Logistic regression was selected as the primary analytical tool because it provides straightforward and clinically interpretable odds ratios (ORs) for the overall risk within a fixed 5-year time horizon. The final model included covariates selected based on clinical relevance and univariate associations (*P_FDR* < 0.1), including age at ESKD diagnosis, sex, baseline estimated glomerular filtration rate (eGFR). A forward likelihood ratio (LR) method was employed for the multivariable selection process. Determine the predictive validity of candidate risk factors through ROC curve analysis. Statistical significance was defined by *P_FDR* values of <0.05.

In the logistic regression, continuous variables were converted into binary categorical variables as follows: elevated cTnI was defined as ≥0.04 μg/L, elevated NT-proBNP was defined as ≥300 pg/mL, and low IgG was defined as <7 g/L.

## 3. Results

### 3.1. Study Population

**Figure 1:**
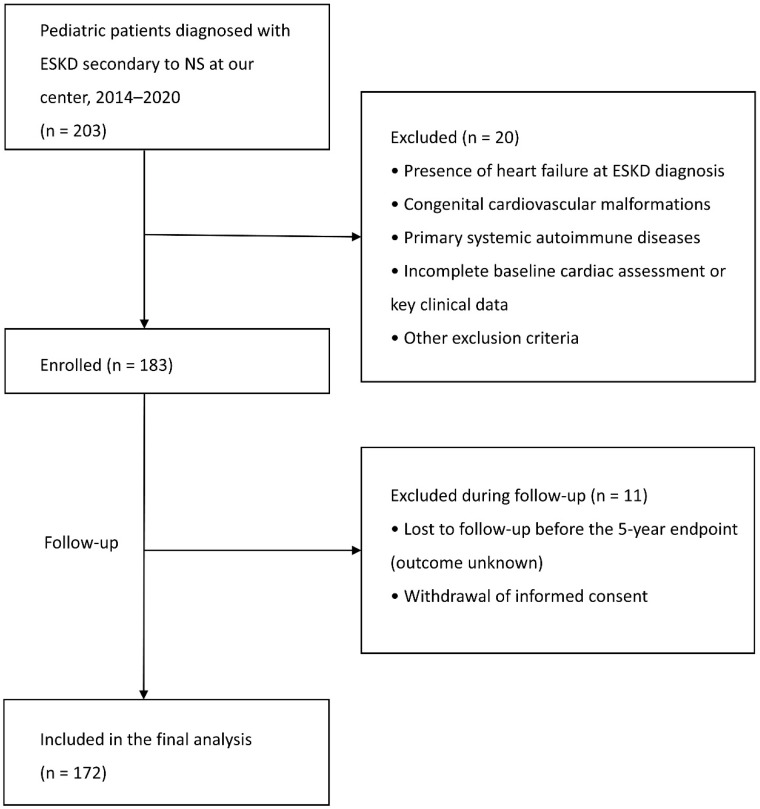
Study Population Flow Diagram.

This study included 172 patients with initial diagnosis of ESKD associated with SRNS (Figure 1). All patients received regular RRT and completed a minimum 5-year follow-up period or were followed until death. At the 5-year follow-up endpoint, 119 patients (69.2%) remained alive, while mortality occurred in 53 patients (30.8%). Baseline demographic characteristics including gender, age, and eGFR showed no statistically significant differences between survivor and non-survivor groups. During the 5-year follow-up period following initial ESKD diagnosis, 45 patients (26.2%) developed HF. Notably, 27 patients (15.7%) experienced HF onset within the first year post-ESKD diagnosis, representing an aggressive phenotype of de novo HF development. This early-onset HF subset was associated with a more rapid progression to cardiovascular complications compared to patients with later HF development.

### 3.2. Factors Associated with All-Cause Death of Pediatric ESKD

**Figure 2:**
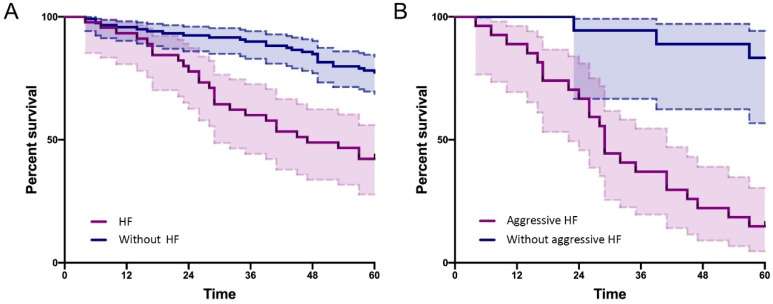
Survival curve of pediatric ESKD patients with and without HF. (**A**). Patients with HF had a higher incidence of death during the five-year follow-up. (**B**). Five-year survival trajectory of patients stratified by the development of aggressive HF.

To identify potential risk factors associated with mortality in pediatric ESKD patients, we analyzed initial laboratory parameters and adverse clinical events occurring during the follow-up period. Univariable analysis revealed several parameters significantly associated with all-cause mortality in pediatric ESKD patients (Table 1). The development of HF following ESKD diagnosis indicated a strong association with adverse clinical outcomes (*p* < 0.0001), representing a positive predictor of all-cause mortality. Kaplan–Meier survival analysis was performed to evaluate the impact of concurrent HF on patient survival (Figure 2A). The survival curve indicated a marked reduction in patient survival following HF onset (log-rank test, *p* < 0.0001). The 5-year survival rate for pediatric ESKD patients with concurrent HF was 42.2%, compared to 78.7% for patients who remained free from HF during the follow-up period. This substantial difference underscores the critical importance of HF prevention and management in improving survival outcomes among pediatric ESKD patients. Comparative survival analysis between aggressive and non-aggressive HF phenotypes revealed distinct prognostic implications (Figure 2B). Patients with rapid-onset aggressive HF indicated significantly worse survival outcomes and increased all-cause mortality risk (log-rank test, *p* < 0.0001), characterized by markedly reduced survival probability. The median survival time for patients in the HF group was 34 months (95% CI: 22–47 months), whereas the median survival for the group without HF was not reached during the 60-month follow-up period (the 5-year survival rate was 78.7%).

### 3.3. Factors Associated with HF of Pediatric ESKD

Given that HF emerged as the predominant predictor of adverse outcomes, we conducted a comprehensive analysis of baseline clinical parameters and early-stage ESKD characteristics to identify potential predictive factors. This analysis encompassed laboratory test results, clinical manifestations associated with renal disease, and cardiovascular assessments at the time of initial ESKD diagnosis (Table 2). Baseline demographic characteristics including gender, age, and initial eGFR at ESKD diagnosis showed no significant differences between pediatric patients who subsequently developed HF and those who remained HF-free. Similarly, metabolic parameters, inflammatory markers, and infection-related laboratory values indicated no significant differences between the two groups. This finding was consistent with comparable rates of documented clinical history including recurrent pulmonary infections, secondary hyperparathyroidism, persistent anemia, treatment-resistant metabolic acidosis, and hyperuricemia prior to reaching ESKD. Among the laboratory parameters examined, only serum IgG was significantly decreased in ESKD patients who subsequently developed HF (*p* = 0.042), suggesting a potential association between immune dysfunction and cardiac complications. Clinical cardiovascular risk factors including hypertension, hypertensive encephalopathy, thrombotic events, and family history of cardiovascular disease showed no significant differences between groups prior to HF development. Notably, significant elevations in cardiac biomarkers were observed at initial ESKD diagnosis in patients who subsequently developed HF. Cardiac troponin I (cTnI) levels were significantly elevated (*p* < 0.001), as were N-terminal pro-B-type natriuretic peptide (NT-proBNP) concentrations (*p* < 0.001), indicating early myocardial injury and stress. Furthermore, the majority of ESKD patients who later developed HF already had documented arrhythmias (82.2%) at the time of initial ESKD diagnosis, suggesting that subclinical cardiac dysfunction precedes clinically apparent HF in this population.

To identify early echocardiographic parameters associated with subsequent HF development, we conducted comprehensive cardiac assessments at the time of initial ESKD diagnosis, focusing on cardiac chamber dimensions and valvular function (Table 3). Initial echocardiographic evaluation revealed several cardiac abnormalities significantly associated with future HF development. Left ventricular dilation (*p* < 0.001), global cardiac enlargement (*p* = 0.002), and ventricular hypertrophy (*p* = 0.006) were significantly more prevalent in patients who subsequently developed HF. Additionally, valvular regurgitation across multiple sites showed strong associations with HF risk, including aortic regurgitation (*p* < 0.001), mitral regurgitation (*p* < 0.001), and tricuspid regurgitation (*p* < 0.001).

Multivariate logistic regression analysis was performed to identify independent predictors of HF development. The following parameters emerged as independent risk factors: left ventricular dilation (OR = 23.669, 95% CI: 2.876–194.827), aortic regurgitation (OR = 3.851, 95% CI: 1.205–12.304), mitral regurgitation (OR = 4.904, 95% CI: 1.197–20.085), tricuspid regurgitation (OR = 3.796, 95% CI: 2.048–13.583), arrhythmia (OR = 2.951, 95% CI: 1.260–6.912), and elevated cardiac troponin I (OR = 6.786, 95% CI: 2.326–19.799). ROC curve analysis was conducted to evaluate the discriminative performance of the identified independent predictors. The area under the curve (AUC) values indicated varying predictive capabilities: left ventricular dilation showed excellent discriminative ability (AUC = 0.850, 95% CI: 0.786–0.915), followed by mitral regurgitation with good predictive value (AUC = 0.807, 95% CI: 0.724–0.889). Cardiac troponin I (AUC = 0.723, 95% CI: 0.597–0.848) and aortic regurgitation (AUC = 0.692, 95% CI: 0.597–0.816) suggested moderate predictive performance, while arrhythmia showed fair discriminative ability (AUC = 0.632, 95% CI: 0.517–0.747). These findings suggest that comprehensive echocardiographic assessment at ESKD diagnosis can effectively identify patients at high risk for HF development, potentially enabling early intervention strategies to improve cardiovascular outcomes in this vulnerable population.

## 4. Discussion

The global burden of CKD is substantial. According to the World Health Organization’s Global Health Estimates, CKD was attributed to 864,226 deaths in 2012, accounting for 1.5% of global mortality. In our pediatric ESKD cohort, the mortality rate reached 30.8%, with a heart failure (HF) incidence of 26.2%. Importantly, HF was independently associated with mortality, consistent with prior evidence that cardiovascular complications are the leading cause of death in children with ESKD [20,21]. Unlike adult populations, in which cardiorenal progression is typically driven by cumulative traditional cardiovascular risk factors such as hypertension, diabetes, and dyslipidemia [1], our pediatric cohort demonstrated a distinct risk pattern. A history of hypertension was not a significant predictor of HF (*P_FDR* = 0.985), highlighting ongoing uncertainty regarding the prognostic role of conventional risk factors in children [17,18]. Our findings suggest that once pediatric patients progress to ESKD, established structural cardiac abnormalities may outweigh earlier clinical risk profiles. This challenges the direct extrapolation of adult risk prediction models to children and supports a management paradigm centered on early detection of subclinical cardiac remodeling.

The pathophysiology of cardiorenal syndrome involves multiple interconnected mechanisms. These include activation of the renin–angiotensin–aldosterone system (RAAS) and sympathetic nervous system (SNS), hemodynamic abnormalities, inflammatory and immune responses, oxidative stress, and apoptosis [22,23,24,25,26,27]. In chronic HF, elevated cardiac troponin levels indicate cardiomyocyte injury or necrosis [28]. Although cTnI elevation in CKD may partly reflect reduced renal clearance, its substantially higher levels in the HF group, together with comparable eGFR between groups, support a true signal of myocardial injury. Moreover, current guidelines recognize that even modest troponin elevations in CKD may independently predict cardiovascular risk [1,29]. In multivariable analysis, cTnI remained an independent predictor of HF, whereas NT-proBNP lost significance. This divergence may reflect differences in biological specificity. cTnI directly indicates cardiomyocyte injury [30], whereas NT-proBNP primarily reflects ventricular wall stress, which is closely linked to structural changes such as left ventricular dilation and valvular regurgitation. Therefore, when these structural parameters were incorporated into the multivariate model, the predictive information carried by NT-proBNP may have been encompassed by these more fundamental morphological changes, thereby losing its independent predictive value [31,32]. This suggests that, in pediatric ESKD patients, early cardiac structural remodeling may be a critical step in initiating cardiac functional deterioration, while elevated cTnI may indicate that myocardial injury has progressed to a detectable stage with independent prognostic significance.

Left ventricular dilation results from the combined effects of increased cardiac preload, oxidative stress, xanthine oxidase activation, RAAS-induced hyperaldosteronism, and increased arterial stiffness [33]. Paoletti et al. [34] demonstrated that left ventricular hypertrophy in CKD patients serves as a powerful predictor of adverse cardiovascular outcomes, correlating with systolic and/or diastolic dysfunction and increased arrhythmia risk. Fluid overload secondary to renal dysfunction increases cardiac preload and decompensated left ventricular hypertrophy leads to cardiac dilation. The myocardial remodeling associated with cardiac dilation increases oxygen demand and creates relative myocardial ischemia, establishing a vicious cycle [22,25]. Cardiac dilation also results in functional mitral regurgitation, leading to impaired biventricular function. A significant finding of our study is that cardiovascular structural changes (left heart dilation and valvular regurgitation) constitute risk factors for CRS development, which was significantly associated with all-cause mortality in pediatric ESKD patients. This finding is consistent with the established paradigm in adult studies where cardiovascular disease represents the leading cause of death in ESKD patients. An important consideration is that left heart enlargement present at the time of initial ESKD diagnosis, serving as a marker of cardiac structural remodeling, can independently predict the subsequent development of systolic dysfunction meeting EF/FS criteria over time. This aligns with the consensus in cardiovascular pathophysiology that cardiac structural changes often precede functional failure, further supporting the clinical significance of early identification of cardiac structural abnormalities in preventing the progression of heart failure. Additionally, in our cohort, these parameters were measured at the baseline visit when patients were free of clinical HF by standard criteria. Their strong association with future HF events underscores that they represent advanced, subclinical myocardial remodeling. These findings emphasize that the detection of such structural abnormalities in children with ESKD, even in the absence of fulminant symptoms, should trigger heightened clinical concern and consideration for early intervention, as these patients are at imminent risk of progressing to symptomatic heart failure. Therefore, early identification of cardiac injury and morphological changes should prompt initiation of anti-remodeling or anti-HF therapies, which may improve outcomes in ESKD patients.

The pathogenesis of cardiorenal syndrome in pediatric ESKD patients involves complex bidirectional interactions between cardiac and renal dysfunction. Recent evidence suggests that uremic toxins, including indoxyl sulfate and p-cresyl sulfate, contribute to cardiovascular dysfunction through endothelial damage and increased oxidative stress [35]. Furthermore, mineral and bone disorder associated with CKD, characterized by hyperphosphatemia and secondary hyperparathyroidism, accelerates vascular calcification and cardiac dysfunction [36]. The role of inflammation in pediatric CRS deserves particular attention. Chronic inflammation in ESKD patients leads to elevated levels of pro-inflammatory cytokines, including interleukin-6 and C-reactive protein, which directly impair cardiac function and promote atherosclerosis [37]. The observed association between low IgG levels and the development of CRS in our study may reflect not only an increased susceptibility to infection but also protein-energy wasting and chronic inflammation, which was considered to be associated with HF. Furthermore, it might be related to cumulative immunological damage resulting from long-term prior immunosuppressive therapy. Future prospective studies should meticulously record the cumulative exposure to immunosuppressive agents to elucidate their independent impact on cardiovascular outcomes following the onset of ESKD.

The limitations of this study should be interpreted within the context of its retrospective design and clinical setting. Firstly, the single-center, retrospective nature of the study inherently introduces selection bias and restricts the generalizability of our findings. Our cohort was sourced exclusively from a tertiary referral center in southwestern China, which may not fully capture the diverse racial, geographic, or socioeconomic characteristics of the broader pediatric ESKD population. Future prospective, multicenter studies are essential to validate the biological universality of our results across different demographic and medical backgrounds. Secondly, the advanced disease stage of our cohort at presentation precluded a unified histopathological classification. Many patients are already in the ESKD stage with extensive renal fibrosis when they seek medical attention, and therefore do not undergo diagnostic renal biopsy. Consequently, despite using rigorous clinical criteria to exclude secondary or syndromic etiologies, the lack of comprehensive genetic data and specific histological subtypes prevents us from definitively linking distinct underlying SRNS pathologies to specific cardiovascular outcomes. Thirdly, reliance on historical medical records resulted in unavoidable missing data and restricted the granularity of our clinical phenotyping. Several potentially relevant variables, including β2-microglobulin, cystatin C, specific proteinuria values, and actual left ventricular mass could not be systematically analyzed. Furthermore, our dataset lacked granular sub-categorizations for clinical events, such as specific arrhythmia phenotypes. We also did not systematically capture post-ESKD residual urine volume, urinary protein excretion, or the potential therapeutic benefits of diuretic administration in this cohort. Additionally, while standard coagulation markers (e.g., antithrombin III, D-dimer) were monitored, the absence of comprehensive hematological profiling prevents us from concluding whether specific inherited or acquired coagulopathies independently drive heart failure risk. Future longitudinal studies must integrate these granular metrics to elucidate the precise mechanisms of cardiorenal crosstalk. Finally, our statistical models face inherent methodological challenges due to the relatively small number of heart failure events relative to the predictors evaluated. This imbalance likely accounts for the wide confidence interval and extremely high odds ratio observed for left ventricular dilation, indicating potential model instability or overfitting. Furthermore, the tight pathophysiological correlation between left ventricular dilation and valvular regurgitation inevitably introduces multicollinearity when both are included in multivariable models, potentially destabilizing their independent effect estimates. Therefore, the specific quantitative point estimates should be interpreted with caution. Nevertheless, these statistical limitations do not negate our core clinical insight: the detection of early subclinical cardiac structural abnormalities, whether isolated or clustered, serves as a highly robust, early warning signal. These structural changes underscore the critical value of systematic baseline echocardiographic screening to guide preemptive clinical interventions in pediatric ESKD.

## 5. Conclusions

The findings of this study highlight the importance of cardiovascular monitoring in the clinical management of pediatric ESKD patients and suggest potential directions for optimization. Based on our observations, it may be reasonable to consider a comprehensive baseline assessment for all children newly diagnosed with ESKD during initial hospitalization. This assessment should include biomarker testing such as cardiac troponin I, electrocardiography to detect arrhythmias, and echocardiography to evaluate cardiac structure and function. Furthermore, in children with identified risk factors, periodic monitoring, for example, performing biomarker tests every 3 months and echocardiograms every 6 months, could facilitate earlier detection of cardiac functional deterioration. The potential benefits of such early monitoring and intervention include delaying or preventing the onset of heart failure, improving patients’ quality of life, and contributing to a reduction in the high mortality rate observed in this vulnerable population.

## Figures and Tables

**Table 1:** Baseline characteristics and univariate analysis of factors associated with all-cause mortality in pediatric ESKD.

Variable	Survival Group (n = 119)	Death Group (n = 53)	*P_FDR*
Female/male (n)	52/67	24/29	0.916
Age (year)	10.4 (3.5)	9.3 (3.9)	0.230
eGFR (mL/(min·1.73 m^2^))	9.5 (3.6)	10.4 (3.5)	0.401
EPO (mIU/mL)	64.7 (167.5)	34.3 (49.9)	0.755
K^+^ (mmol/L)	4.3 (1.0)	4.5 (0.9)	0.798
CL^−^ (mmol/L)	104.9 (9.5)	103.9 (9.3)	0.848
Ca^2+^ (mmol/L)	0.8 (0.2)	0.9 (0.3)	0.598
P^−^ (mmol/L)	2.7 (1.2)	2.6 (1.0)	0.947
GLU (mmol/L)	6.4 (2.5)	6.3 (1.6)	0.848
EOS (×10^9^/L)	2.3 (2.6)	1.9 (1.8)	0.775
NEUT (×10^9^/L)	6.0 (3.5)	6.2 (4.7)	0.848
MCH (pg)	28.3 (2.3)	27.8 (2.5)	0.353
PLT (×10^9^/L)	217.2 (94.9)	226.9 (117.5)	0.848
GLB (g/L)	26.8 (6.0)	24.7 (6.4)	0.170
AST (U/L)	55.3 (119.1)	68.2 (242.8)	0.331
ALT (U/L)	43.7 (98.1)	43.7 (148.7)	0.308
cTnI (μg/L)	0.31 (0.82)	0.27 (0.43)	0.848
CK-MB (U/L)	3.6 (6.7)	11.7 (32.6)	0.308
NT-proBNP (pg/mL)	77.0 (13.6)	57.9 (41.3)	0.447
Mb (ng/dL)	274.3 (359.7)	268.6 (396.0)	0.598
INR	1.2 (0.3)	1.3 (0.6)	0.947
DDI (mg/L)	3.5 (4.7)	2.7 (3.3)	0.731
FDP (mg/L)	10.7 (13.7)	8.7 (9.2)	0.947
ATIII (%)	103.3 (22.3)	96.0 (24.0)	0.370
CD3^+^CD8^+^	29.3 (8.9)	27.0 (10.9)	0.659
hs-CRP (mg/L)	8.0 (16.6)	4.2 (6.5)	0.433
IgE (IU/mL)	158.6 (366.1)	99.0 (195.8)	0.659
IgG (g/L)	8.2 (3.7)	7.1 (4.0)	0.353
Heart failure	19 (16.0%)	26 (49.1%)	<0.001
Aggressive heart failure	4 (3.4%)	23 (43.4%)	<0.001
Recurrent edema	55 (46.2%)	34 (64.2%)	0.170
Hypertension	70 (58.8%)	33 (62.3%)	0.848
Hypertensive encephalopathy	12 (10.1%)	5 (9.4%)	0.947
Initial arrhythmia records	49 (41.2%)	37 (69.8%)	0.916
Secondary hyperparathyroidism	70 (58.8%)	25 (47.2%)	0.331
Recurrent pulmonary infection	73 (61.3%)	34 (64.2%)	0.848
Persistent anemia	108 (90.8%)	47 (88.7%)	0.848
Thrombosis	6 (5.0%)	6 (11.3%)	0.308
Resistant metabolic acidosis	53 (44.5%)	21 (39.6%)	0.775
Hyperuricemia	37 (31.1%)	17 (32.1%)	0.947
Family history of renal disease	43 (36.1%)	21 (39.6%)	0.848

Continuous data were presented as mean (SD), while stratification variable was presented as n (%). GFR, glomerular filtration rate; EPO, erythropoietin; GLU, glucose; EOS, eosinophil count; NEUT, neutrophil count; MCH, mean corpuscular hemoglobin; PLT, platelet count; GLB, globulin; AST, aspartate aminotransferase; ALT, alanine aminotransferase; cTnI, cardiac troponin I; CK-MB, creatine kinase-MB; NT-proBNP, N-terminal pro-brain natriuretic peptide; Mb, myoglobin; INR, international normalized ratio; DDI, D-dimer; FDP, fibrin degradation products; ATIII, antithrombin III; hs-CRP, high-sensitivity C-reactive protein.

**Table 2:** Baseline characteristics and univariate analysis of factors associated with heart failure development in pediatric ESKD.

Variable	Non-HF Group (n = 127)	HF Group (n = 45)	*P_FDR*
Female/male (n)	54/73	23/22	0.669
Age (year)	10.2 (3.5)	9.2 (4.4)	0.328
eGFR (mL/(min·1.73 m^2^))	9.9 (3.7)	9.5 (3.4)	0.784
EPO (mIU/mL)	48.4 (116.9)	101.6 (262.1)	0.991
K^+^ (mmol/L)	4.4 (1.0)	4.3 (0.9)	0.784
CL^−^ (mmol/L)	105.1 (8.9)	102.6 (11.4)	0.711
Ca^2+^ (mmol/L)	0.8 (0.3)	0.9 (0.3)	0.649
P^−^ (mmol/L)	2.6 (1.0)	2.8 (1.5)	0.881
GLU (mmol/L)	6.4 (2.5)	6.2 (1.2)	0.769
EOS (×10^9^/L)	2.3 (2.5)	1.5 (1.4)	0.581
NEUT (×10^9^/L)	6.1 (4.0)	5.8 (3.3)	0.991
MCH (pg)	28.1 (2.5)	28.6 (1.6)	0.691
PLT (×10^9^/L)	222.9 (100.1)	205.9 (113.8)	0.328
GLB (g/L)	26.5 (6.5)	24.4 (4.4)	0.141
AST (U/L)	58.8 (176.3)	59.4 (94.4)	0.172
ALT (U/L)	40.8 (114.5)	57.4 (118.8)	0.328
cTnI (μg/L)	0.21 (0.73)	1.43 (1.95)	<0.001
CK-MB (U/L)	5.4 (18.0)	6.1 (7.9)	0.328
NT-proBNP (pg/mL)	63.9 (50.1)	379.2 (62.3)	<0.001
Mb (ng/dL)	270.4 (387.2)	281.2 (292.1)	0.748
INR	1.2 (0.4)	1.3 (0.5)	0.341
DDI (mg/L)	3.2 (4.4)	3.6 (4.4)	0.296
FDP (mg/L)	10.0 (12.9)	10.5 (11.7)	0.328
ATIII (%)	101.8 (23.6)	99.2 (20.7)	0.821
CD3^+^CD8^+^	29.6 (9.8)	24.4 (7.2)	0.296
hs-CRP (mg/L)	6.6 (14.9)	7.3 (7.8)	0.341
IgE (IU/mL)	145.4 (339.4)	110.0 (212.5)	0.649
IgG (g/L)	8.0 (3.9)	5.7 (2.8)	0.042
Hypertension before ESKD	76 (59.8%)	27 (60.0%)	0.985
Hypertensive encephalopathy before ESKD	11 (8.66%)	8 (17.7%)	0.219
Arrhythmias before ESKD	46 (36.2%)	37 (82.2%)	<0.001
Secondary hyperparathyroidism before ESKD	70 (55.1%)	25 (55.6%)	0.985
Recurrent pulmonary infection before ESKD	77 (60.6%)	32 (71.1%)	0.368
Persistent anemia before ESKD	113 (88.9%)	42 (93.3%)	0.624
Thrombosis before ESKD	10 (7.8%)	2 (4.4%)	0.649
Resistant metabolic acidosis before ESKD	54 (42.5%)	20 (44.4%)	0.931
Hyperuricemia before ESKD	40 (31.5%)	13 (28.9%)	0.931
Family History of CVD	44 (34.6%)	22 (48.9%)	0.219

Continuous data were presented as mean (SD), while stratification variable was presented as n (%). GFR, glomerular filtration rate; EPO, erythropoietin; GLU, glucose; EOS, eosinophil count; NEUT, neutrophil count; MCH, mean corpuscular hemoglobin; PLT, platelet count; GLB, globulin; AST, aspartate aminotransferase; ALT, alanine aminotransferase; cTnI, cardiac troponin I; CK-MB, creatine kinase-MB; NT-proBNP, N-terminal pro-brain natriuretic peptide; Mb, myoglobin; INR, international normalized ratio; DDI, D-dimer; FDP, fibrin degradation products; ATIII, antithrombin III; hs-CRP, high-sensitivity C-reactive protein; CVD, cardiovascular disease.

**Table 3:** Initial cardiac assessments in predicting HF in Children with ESKD.

Variable	Univariable Analysis			Logistic Analysis	
	Non-HF Group (n = 127)	HF Group (n = 45)	*P_FDR*	OR (95% CI)	*p*
Left ventricular dilation	33 (26.0%)	43 (95.6%)	<0.001	23.669 (2.876–194.827)	0.003
Whole heart enlargement	4 (3.1%)	12 (26.7%)	0.002	2.611 (0.818–17.011)	0.072
Ventricular hypertrophy	39 (30.7%)	25 (55.6%)	0.006	1.783 (0.529–11.714)	0.278
Pulmonary artery dilation	2 (1.6%)	3 (6.7%)	0.124		
Ascending aorta dilation	2 (1.6%)	0 (0.0%)	0.437		
Aortic valves regurgitation	12 (9.4%)	22 (48.9%)	<0.001	3.851 (1.205–12.304)	0.023
Mitral valves regurgitation	35 (27.6%)	40 (88.9%)	<0.001	4.904 (1.197–20.085)	0.027
Tricuspid regurgitation	39 (30.7%)	28 (62.2%)	<0.001	3.796 (2.048–13.583)	0.021
Stenosis of pulmonary artery valves	3 (2.7%)	3 (6.7%)	0.124		
Pericardial effusion	28 (22.0%)	11 (24.4%)	0.741		
Dilation of coronary arteries	2 (1.6%)	1 (3.7%)	0.437		
Pulmonary hypertension	3 (2.4%)	3 (6.7%)	0.237		

## Data Availability

All the data have been presented in the manuscript. Other data sets used in this study are available from the corresponding author upon reasonable request.

## Supplementary Materials

The following supporting information can be downloaded at: https://www.mdpi.com/article/10.3390/medicina62061131/s1, Table S1: Number of missing key variables.
