# Supplementary material for: Predictors of Heart Failure in Pediatric Patients with End-Stage Kidney Disease Secondary to Nephrotic Syndrome
Source: Medicina (Kaunas). 2026 Jun 10;62(6):1131. doi: 10.3390/medicina62061131 (PMC13303514; doi:10.3390/medicina62061131)
Supplement: Supplementary file 1 [file medicina-62-01131-s001.zip › medicina-4263697-supplementary.pdf]

Supplementary Table S1. Number of missing key variables.

Variable	Missing Cases	Missing Ratio
K^+ (mmol/L)	3	1.7%
CL^- (mmol/L)	3	1.7%
Ca^{2+} (mmol/L)	3	1.7%
cTnI (μg/L)	6	3.5%
CK-MB (U/L)	7	4.0%
NT-proBNP (pg/ml)	5	2.9%
DDI (mg/L)	8	4.7%
FDP (mg/L)	8	4.7%
